# Endovascular treatment of a mycotic aneurysm in an immunocompromised child with acute leukemia—case report and review of the literature

**DOI:** 10.3389/fped.2023.1136647

**Published:** 2023-09-28

**Authors:** Sabine Daphne Diepenbruck, Andre Jakob, Nikolaus Alexander Haas, Guido Mandilaras

**Affiliations:** Division of Pediatric Cardiology and Pediatric Intensive Care, University Hospital of Munich (LMU), Munich, Germany

**Keywords:** endovascular treatment, thoracic mycotic aneurysm, covered stent implantation, leukemia, bacteremia

## Abstract

Mycotic aneurysms are dilatations of an artery, a rare but severe complication arising from infectious obliteration of the vessel wall. Thoracic aneurysms often present with unspecific symptoms and multiple diagnostic and therapeutic challenges. In an advanced state, they have an increased risk of perforation and a high mortality rate. The surgical therapeutic approach has a high perioperative complication rate. In this study, we report a case of a thoracic mycotic aortic aneurysm in an immunocompromised pediatric patient caused by *Klebsiella pneumoniae* bacteremia. A combination of prompt antibacterial treatment and minimally invasive stent implantation showed a good outcome, avoiding possible severe surgical problems.

## 1. Introduction

Mycotic arterial aneurysms are localized dilatations of an artery caused by infectious destruction of the vessel wall. They may occur due to pre-existing arterial wall alterations such as atherosclerosis becoming secondarily infected or due to primary (embolic) infections of the arterial wall, resulting in the development of a new aneurysm. The risk factors are bacteremia, arterial lesions, and a compromised immune system. In pediatrics, mycotic aneurysms of the aorta are an extremely rare disease with a high mortality rate caused by spontaneous rupture (1–3).

In this study, we report the case of a pediatric patient with an immune system compromised due to chemotherapy who developed a mycotic aneurysm of the thoracic aorta within the scope of a severe septic bacterial infection.

## 2. Case report

An 11-year-old boy was transferred to the Department of Pediatric Cardiology and Intensive Care after an incidental radiologic diagnosis of a mycotic aneurysm of the thoracic aorta. The patient had been diagnosed with acute leukemia (pre-T-ALL) 9 months prior to the incident and had been put on a chemotherapeutic regimen since then. During the course of the chemotherapeutic treatment, he suffered repetitive infections such as recurring *Clostridium difficile* enteritis, an extensive perianal abscess, severe osteomyelitis, and esophageal ulceration.

**Figure 1:**
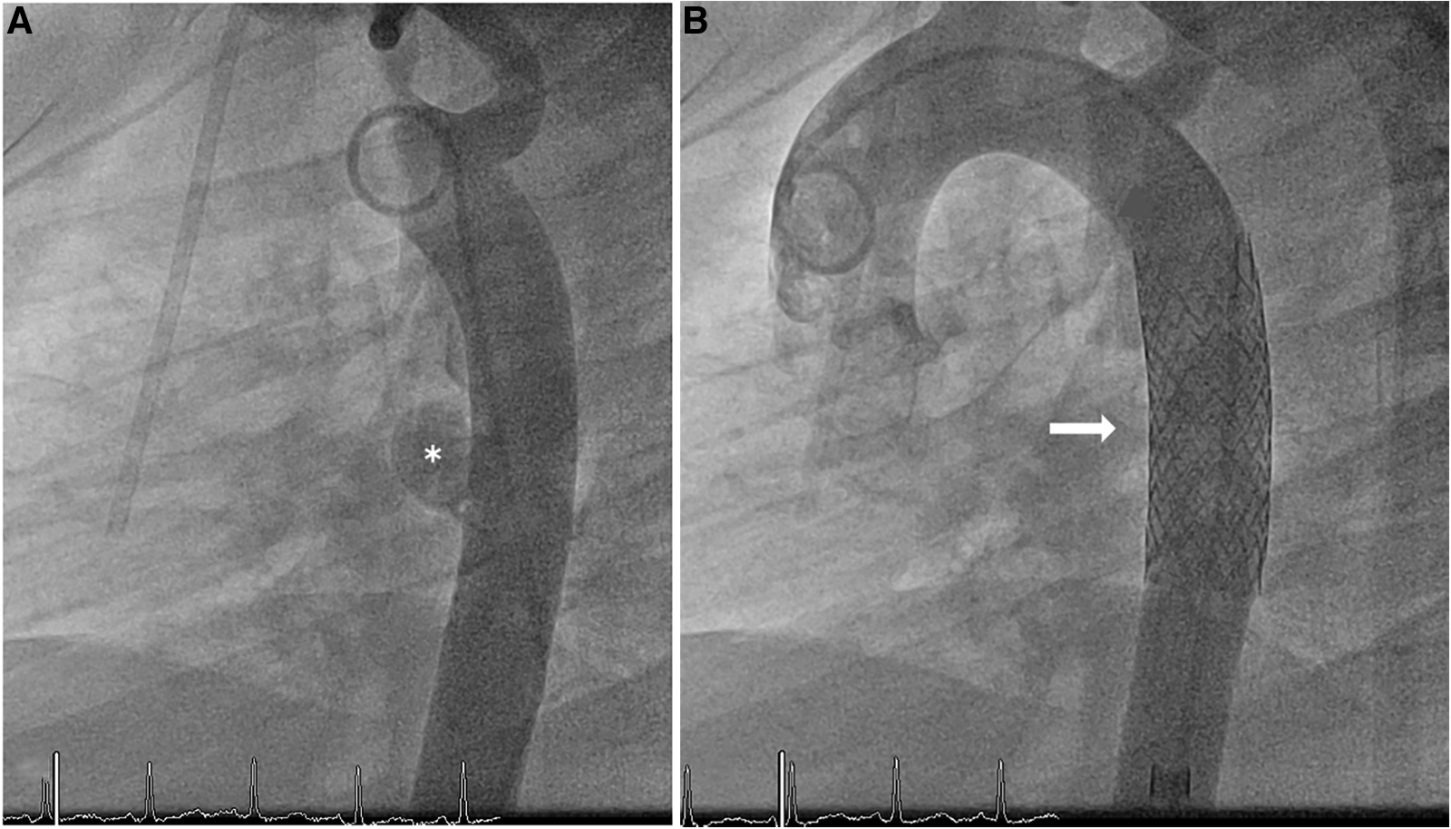
Thoracic CT scan (**A**) pre-intervention, (**B**) post-intervention. * indicates a mycotic aneurysm in the wall of the descending aorta; 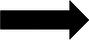 indicates a BeGraft aortic stent.

The present cause of admission was respiratory distress with neutropenia and elevated infection markers. The obtained blood cultures detected *Klebsiella pneumoniae*, and the initially started antibiotic therapy with piperacillin/tazobactam was changed to meropenem. Nonetheless, the patient showed no clinical improvement with persistent tachypnea and developed a pleural effusion. A thoracic CT scan performed to rule out pulmonary aspergillosis revealed an aortic aneurysm of the thoracic aorta with a maximal diameter of 13 mm (Figure 1).

Because of the size of the aneurysm (13 mm × 13 mm) and its increased risk of sudden rupture, the patient was transferred to the pediatric cardiology and intensive care unit. Following a discussion by a multidisciplinary team, an interventional endovascular treatment instead of a surgical approach was favored because of the unstable condition of the patient, with ongoing immunosuppression and inadequate wound healing resulting from the chemotherapy.

Prior to the intervention, the antibacterial treatment was adjusted to intravenous rifampicin and moxifloxacin after 4 days of persistent fever, as determined by the resistogram. To decrease the risk of reinfection due to a contingent infection of the implanted stent, the treatment was continued for 10 days until the catheterization was performed. Eventually, a covered BeGraft stent (16/48) was successfully implanted over the aortic aneurysm in an analgosedation without any adverse events—the diameter of the transverse arch was measured at 16 mm, while the descending aorta exhibited a diameter of 15 mm. Post-interventional prophylactic anticoagulation with heparin (200I.E./kg/day) was performed for 48 h. Furthermore, long-term therapy with acetylsalicylic acid 5 mg/kg was initiated. The young patient was administered the aforementioned antibiotic regimen over a duration spanning 2 months, with 26 days of intravenous administration. Subsequently, the therapeutic approach transitioned to a protracted oral prophylactic regimen in strict adherence to the protocols established by the Department of Pediatric Oncology.

**Figure 2:**
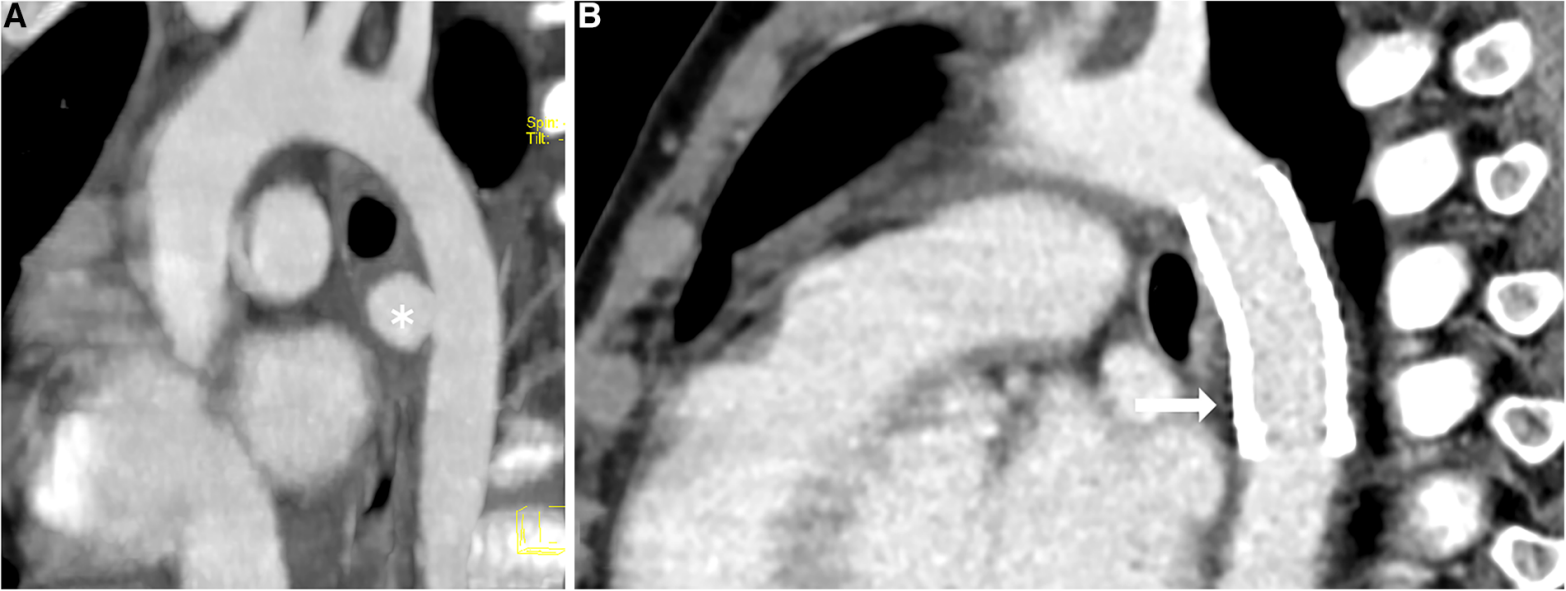
Angiography of the aorta (**A**) pre-intervention, (**B**) post-intervention. Demonstration of the mycotic aneurysm (*) in the wall of the descending aorta; no evidence of extravasation after the implantation of the covered BeGraft aortic stent (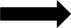).

Follow-up controls, including a thoracic CT scan done 2 weeks after the intervention, showed an excellent postinterventional result with correct positioning of the stent and no further enlargement of the aneurysm (Figure 2). Fortunately, the patient developed no signs of in-stent infection and was able to complete his chemotherapy regimen, and (18 months later) he is still in remission. Cardiological follow-ups at 1, 3, 6, and 12 months after the intervention also indicated no in-stent stenosis and no further aneurysmatic dilation. Further imaging is planned within the scope of the oncological follow-ups. Furthermore, yearly cardiological assessments are being planned to detect a possible indication for stent dilation caused by the patient's growth.

## 3. Discussion

A mycotic aneurysm, also called an “infected aneurysm”, is a dilatation of an artery caused by a bacterial, viral, or fungal infection of the vessel wall (1). The most prevalent pathogens are *Salmonella*, *Staphylococci*, *Streptococci*, and *Klebsiella* species (2). However, there are a variety of different atypical pathogens with regional predominance (3).

The prevalence of mycotic aortic aneurysms in adults is rare (estimated incidence: 0.7%–3% of all aortic aneurysms) (4) and is even less common in minors. Mycotic aneurysms may result due to a hematogenous spread of infection, such as bacteremia, with predisposing arterial lesions and bacterial emboli, or contiguous involvement from an adjacent source of infection, such as osteomyelitis or pneumonia. Predisposing risk factors in adults are atherosclerosis, hypertension, diabetes, and immunosuppression, whereas the main risk factors in children are reported cases of endocarditis, pericarditis, congenital heart defects, such as aortic coarctation, aortic or cardiac surgery, and an (indwelling) arterial catheter(5–7).

The most commonly described symptoms can be rather unspecific and include fever, pain, and leukocytosis (8). Hence, diagnosis is often delayed or incorrect. Diagnostic approaches include a positive blood culture in combination with radiologic findings. Contrast-enhanced CT, MRI, or PET-CT are the imaging modalities of choice to detect arterial aneurysms, as ultrasound, which depends on the location of the aneurysm, may be unreliable (9). Furthermore, a tissue biopsy of the infected arterial wall can be useful to confirm the diagnosis and detect the pathogen. However, these might be difficult to obtain, especially when performing an endovascular repair, and result in a reliable positive culture only in 50%–70% of cases (10, 11).

Infected aortic aneurysms may show a rapid increase in diameter, resulting in an increased risk of rupture. In the adult population, the mortality rates caused by mycotic aneurysms were described as being as high as 75% if untreated (8). Furthermore, they carry with them an increased risk of septic deterioration. Hence, a quick diagnosis with prompt treatment is essential for patient survival. While open surgery has long been the gold standard for the therapeutic approach, high perioperative complications have led to an increased interest in endovascular repair options. Surgical management includes the debridement and resection of the infected tissue and revascularization using autologous or infection-resistant graft material. While the advantage of this method is infection control with better long-term outcomes (12), it is accompanied by high rates of perioperative mortality and morbidity (4). Hence, endovascular repair is especially useful in high-risk patients in whom open surgery is not an option. It is minimally invasive and easily accessible, and current studies in adults report a good short-term survival benefit without late disadvantages (13, 14) or even comparable long-term outcomes (15). Regardless of the open surgical or endovascular approach, all patients require antibiotic treatment for at least 4–8 weeks, or even lifelong treatment, which is ideally adjusted to microbiological findings. Although this therapeutic approach is well described for adults, only a few case reports with therapeutic strategies have been reported for children (Table 1).

**Table 1:** Literature of primary minimal-invasive/endovascular therapy of mycotic aneurysms in pediatric patients.

	No. of patients	Type of pathology	Type of stent
Gupta et al. (16)	1	Mycotic intracavernous carotid artery aneurysm secondary to post-infective cavernous sinus thrombosis	3.5 mm × 27 mm Aneugraft PCS (ITGI Medical, OR Akiva, Israel)
Quinney et al. (17)	1	Mycotic ascending aortic aneurysm after pericarditis from methicillin-sensitive *Staphylococcus aureus* and attempted surgical repair	26 × 10 Gore TAG (W.L. Gore & Associates, USA) across the aortic arch; 11 × 5 Viabahn (W.L. Gore & Associates, USA) & 10 × 37 ICAST (Atrium Medical, USA) in the innominate artery
Skrabonja-Crespo et al. (18)	1	Ascending aortic pseudoaneurysm after heart transplantation and methicillin-sensitive *S. aureus* sepsis	45 mm Covered CP Stent™ (B. Braun Interventional Systems Inc., USA)
Srivastava et al. (19)	1	Infected pseudoaneurysm of a modified Blalock-Taussig shunt	6 mm × 38 mm Advanta V12 (Atrium Medical, USA)
Sunil et al. (7)	1	Mycotic iliac aneurysm with a history of infective endocarditis	7 mm × 58 mm Lifestream BE stent-graft (Becton Dickinson, UK)
Tomar et al. (20)	1	Mycotic aortic aneurysm after coarctation repair	16-20-93 Endurant II Limb Extension stent graft, (Medtronic, USA)

Literature on primary minimally invasive/endovascular therapy of mycotic aneurysms in pediatric patients.

To date, only a few covered, balloon-expandable stents are available for the management of pediatric patients, and these include the CP-covered stent (8–24 mm), the Advanta V12-covered stent (12–16 mm), the Andratec Optimus PTFE-covered stents, and the BeGraft aortic stent.

Here, we report a rare case of an infected aortic aneurysm in a child without a known cardiac or aortic defect but with ongoing immunosuppression and persistent infection. Immunosuppression poses a risk factor for developing mycotic aneurysms. With an increasing number of immunosuppressive therapies available to treat a diverse spectrum of diseases, awareness of possible fatal complications, including infectious aneurysms, becomes essential. The development of sensitive and specific diagnostics and antibacterial treatments helps prevent these complications. Furthermore, the close collaboration of a multidisciplinary team is pivotal for finding the right therapeutic approach for this group of patients.

## 4. Conclusion

To the best of our knowledge, this is the first case of a thoracic mycotic aortic aneurysm described in an immunocompromised pediatric patient with *K. pneumoniae* bacteremia. Given the high risk of rupture, persistent invasive infections in immunocompromised patients should justify further investigation for early detection of potential infectious aneurysms. An immediate antibiotic treatment, combined with sparing interventional stent implantation, may better the outcome and reduce the poor odds of a potential rupture. The above outlines the fact that there is a need for conducting further clinical trials on interventional treatment of aneurysms and developing covered stents appropriate for use in pediatric patients.

## Data Availability

The original contributions presented in the study are included in the article/Supplementary Material, and further inquiries can be directed to the corresponding author.
